# Light-deformable microrobots shape up for the biological obstacle course

**DOI:** 10.1038/s41377-024-01448-8

**Published:** 2024-05-06

**Authors:** Philip Wijesinghe

**Affiliations:** Centre of Biophotonics, SUPA, School of Physics and Astronomy, University of St Andrews, North Haugh, St Andrews, Fife KY16 9SS UK

**Keywords:** Biophotonics, Optical manipulation and tweezers

## Abstract

*Euglena gracilis* microalga has been transformed into a soft bio-microrobot with light-controlled motion and deformation that can address diverse bio-challenges, such as drug delivery, diseased cell removal, and photodynamic therapy.

Micro and nanorobots are emerging as powerful miniaturized machines that can perform diverse automated and precision tasks in biomedicine with innumerable applications, from targeted drug delivery, microsurgery and localized therapy to multiplexed bio-assembly and cell removal^1^. Because microrobots must operate on the microscopic length scale, they face distinct challenges. Specifically, they require modes of locomotion with remote or efficient power delivery, for instance via magnetic or chemical sources^2^, and that can navigate exceptionally low-Reynold’s number environments where the viscous force greatly exceeds that of inertia^3^. Living tissues pose an even greater challenge because of the need to navigate tight interstitial spaces, for instance in epithelial tissues or dense tumor microenvironments. Naturally, soft bioinspired and biohybrid microrobots have propelled developments in this area by borrowing solutions from the biological world^3^.

In a newly published paper in *Light: Science & Applications*, researchers from Jinan University, China, report on a creative use of the *Euglena gracilis* (EG) microalga as a self-propelled, light-controlled and light-deformable bio-microrobot—“Ebot”^4^. This soft biohybrid microrobot harnesses the natural affinity of EG to seek blue light, the dominant visible wavelength in aquatic environments, through its eyespot and photoreceptor to enact controlled and repeatable locomotion and deformation through complex bio-environments (Fig. 1).

**Fig. 1:**
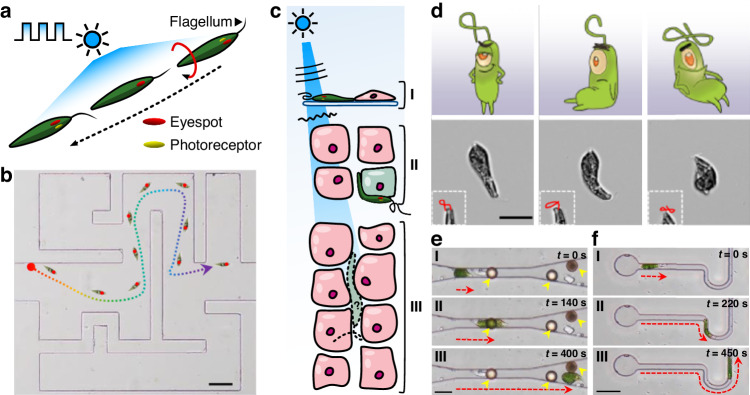
Illustration of the Ebot. **a** *Euglena gracilis* motion in response to light enables (**b**) light-controlled motion through an obstacle course. **c** Precise light control at the (I) surface becomes challenging at (II) shallow depths due to phase aberrations, and is completely scrambled (III) deep in the diffuse regime. **d** Light-controlled deformation with changing illumination intensity. **e**, **f** Phototaxis of Ebots through (**e**) channels with obstacles and (**f**) tight 5-µm channels. Scale bars are 50 µm in (**b**) and 20 µm in (**d**–**f**). Figures are adapted from the original publication^4^

Light is a powerful means of interacting with the microscopic world. The ability to structure and retrieve information with light is typically commensurate with the wavelength, which is sub-micrometer for visible light. This is further assisted by typically facile shaping and manipulation of visible light compared to, for instance, magnetic or acoustic fields^5^. Light can also confer photonic forces to microscopic particles and cells^6^, e.g., using optical tweezers, enabling optical micro-manipulation and assembly^7^ on a smaller scale to that of acoustic forces^8^. Optical forces have been exploited for microrobots^2^, for instance, as a method for inducing a microrobot to perform a particular task^9^. Biohybrid microrobots have also exploited optogenetic control. For instance, transgenic modification can express a photosensitive ion channel in muscle cells achieving optically triggered muscle contraction^10^.

The use of EG microalgae in the context of light-controlled microrobotics is quite remarkable because it exploits and reverse-engineers the natural adaptation of the organism. EG, naturally, are self-propelling, motile organisms that are autofluorescent and respond to light stimuli, especially that of blue light^4^. The motion of an EG is determined by the orientation of its photoreceptors, and the light-shading by an eyespot with respect to the source position of the light (Fig. 1a). The rolling movement of the EG results in a periodic activation of the photoreceptors, while the total light intensity can change the flagella and soft body deformation of the organism. The authors have exploited this natural feedback system to demonstrate precise and repetitive control of EG motion, orientation, as well as control of deformation through tight spaces solely by varying the periodicity and intensity of a light source^4^.

The control of microrobots using light, however, comes with its own challenges. The ability to precisely control a light field rapidly diminishes with depth (Fig. 1c). Optical tweezer methods are typically limited to the surface because they require precise phase and polarization of the light field, which is typically scrambled by scattering tissues^11^. Precise optogenetic activation of individual cells requires the control of high spatial frequencies of light that may be maintained within shallow (~100 µm) sections, which could be further extended with promising adaptive optics approaches^12^. Interestingly, indirect optical trapping through opto-fluidic streaming was also recently demonstrated in *Light, Science & Applications*, overcoming issues with trapping instability^13^. Much deeper, millimeter-deep control could be achieved in the optically diffuse regime if the collective behavior of microrobots can be statistically predicted and controlled. Because of this, the exploitation of naturally adapted motion of microorganisms is of particular interest.

While other microalgae have been employed as microrobots^14,15^, the Ebot is distinct in that it is a soft organism that can support repeatable light-controlled deformation. Because of this, the authors present several compelling demonstrations of Ebots navigating through complex confined microenvironments. Figure 1e, f shows the navigation of the Ebot through confined microchannels with occluding bodies and very thin geometries. This capability is required for many therapeutic and drug delivery applications that must penetrate epithelial cells and into tumor microenvironments. The original publication demonstrates several such applications, including drug delivery, cell removal and photodynamic therapy^4^.

The Ebot adds to the substantive impetus in bioinspired and biohybrid robotics^16^. As seen by the phototaxic motion of EG, biological systems feature feedback mechanisms that can lead to two very valuable properties. Ease of control that is insensitive to minor variations in the environment and that does not require, in this case, precise optical manipulation at depth; and ease of manufacture (and cost) that avoids genetic modification or artificial nanofabrication. While there is much remaining to be explored in their effectiveness at performing tasks at depths in living tissues and the associated biocompatibility, Ebots are certainly shaping up as promising deformable microrobots for the next generation of discoveries.
